# Reduced lung dose during radiotherapy for thoracic esophageal carcinoma: VMAT combined with active breathing control for moderate DIBH

**DOI:** 10.1186/1748-717X-8-291

**Published:** 2013-12-20

**Authors:** Guanzhong Gong, Ruozheng Wang, Yujie Guo, Deyin Zhai, Tonghai Liu, Jie Lu, Jinhu Chen, Chengxin Liu, Yong Yin

**Affiliations:** 1 Department of Radiation Oncology, Shandong Cancer Hospital and Institute, Jinan 250117, China

**Keywords:** Esophageal carcinoma, Radiotherapy, Active breathing control, Volumetric modulated arc radiotherapy

## Abstract

**Background:**

Lung radiation injury is a critical complication of radiotherapy (RT) for thoracic esophageal carcinoma (EC). Therefore, the goal of this study was to investigate the feasibility and dosimetric effects of reducing the lung tissue irradiation dose during RT for thoracic EC by applying volumetric modulated arc radiotherapy (VMAT) combined with active breathing control (ABC) for moderate deep inspiration breath-hold (mDIBH).

**Methods:**

Fifteen patients with thoracic EC were randomly selected to undergo two series of computed tomography (CT) simulation scans with ABC used to achieve mDIBH (representing 80% of peak DIBH value) versus free breathing (FB). Gross tumor volumes were contoured on different CT images, and planning target volumes (PTVs) were obtained using different margins. For PTV_-FB_, intensity-modulated radiotherapy (IMRT) was designed with seven fields, and VMAT included two whole arcs. For PTV_-DIBH_, VMAT with three 135° arcs was applied, and the corresponding plans were named: IMRT_-FB_, VMAT_-FB_, and VMAT_-DIBH_, respectively. Dosimetric differences between the different plans were compared.

**Results:**

The heart volumes decreased by 19.85%, while total lung volume increased by 52.54% in mDIBH, compared to FB (*p* < 0.05). The mean conformality index values and homogeneity index values for VMAT_-DIBH_ (0.86, 1.07) were slightly worse than those for IMRT_-FB_ (0.90, 1.05) and VMAT_-FB_ (0.90, 1.06) (*p* > 0.05). Furthermore, compared to IMRT_-FB_ and VMAT_-FB_, VMAT_-DIBH_ reduced the mean total lung dose by 18.64% and 17.84%, respectively (*p <* 0.05); moreover, the V_5_, V_10_, V_20_, and V_30_ values for IMRT_-FB_ and VMAT_-FB_ were reduced by 10.84% and 10.65% (*p >* 0.05), 12.5% and 20% (*p <* 0.05), 30.77% and 33.33% (*p <* 0.05), and 50.33% and 49.15% (*p <* 0.05), respectively. However, the heart dose-volume indices were similar between VMAT_-DIBH_ and VMAT_-FB_ which were lower than IMRT_-FB_ without being statistically significant (*p* > 0.05). The monitor units and treatment time of VMAT_-DIBH_ were also the lowest (*p* < 0.05).

**Conclusions:**

VMAT combined with ABC to achieve mDIBH is a feasible approach for RT of thoracic EC. Furthermore, this method has the potential to effectively reduce lung dose in a shorter treatment time and with better targeting accuracy.

## Introduction

Esophageal carcinoma (EC) is one of the most common malignant tumor types worldwide, and its incidence continues to rise [1]. Currently, complete surgical resection is the standard treatment for patients with EC who are medically fit [2]. However, chemo-radiotherapy has also achieved promising clinical outcomes for patients with advanced local EC, including squamous cell cancer and adenocarcinoma, as well as for those who are not fit for surgery [2-4]. Radiation therapy (RT) also continues to be a critical component for multimodality systemic treatment of EC [5,6]. However, treatment-related pneumonitis is an acute and toxic side effect that can occur with RT for thoracic EC [7]. Advances have been made in RT technology that can be applied to EC, including the progression from two-dimensional RT to three-dimensional conformal radiotherapy (3D-CRT), intensity modulated radiotherapy (IMRT), intensity proton beam radiotherapy (IMPT), volumetric modulated radiotherapy (VMAT), and helical tomotherapy (HT) [5,8-13]. In particular, IMRT, VMAT, and HT have achieved a more conformal and homogeneous dose distribution with the application of beam intensity modulated technology and a multileaf collimator (MLC) system compared with 3D-CRT. VMAT also represents an extension of IMRT which facilitates synchronized variations in gantry speed, dose rate, and the shape of the MLC and jaws [14,15]. Consequently, VMAT can achieve similar, if not better, dose distribution compared with IMRT with shorter treatment times and fewer monitor units (MUs) [15-19]. The application of VMAT to EC RT has obtained similar results, although a more extensive low dose region is involved which can affect normal tissue [9-13]. However, given the shorter duration of the treatment associated with VMAT and the other technologies available, the damage to normal tissue can be minimized [19].

During RT for thoracic EC, displacement and deformation induced by breath motion are critical factors which affect treatment accuracy and precision. For example, in a report by Yanashita et al. [20], the average displacements in the cranial-caudal (CC) for the upper, middle, and lower thoracic esophagus were 3.2 mm (range, 0.7–5.5 mm), 6.4 mm (range, 1.5–14.5 mm), and 10.3 mm (range, 4.0–16.3 mm), respectively. In comparison, the displacement of the left-right (L-R) and anterior-posterior (AP) regions were smaller. However, application of active breathing control (ABC) has been observed to reduce the motion induced by respiration [21,22]. In addition, lung volume can be effectively increased with DIBH, and this has the benefit of sparing lung tissue during RT. Therefore, the feasibility and dosimetric features of VMAT combined with ABC for mDIBH for RT of thoracic EC were investigated.

## Methods

### Patients

A total of 15 patients (5 females, 10 males) ranging in age from 42–65 y (median, 53.5 y) had pathologically proven thoracic squamous cell EC and were randomly selected from the patients treated at our hospital between March 2010 and December 2011. Cardiopulmonary function for each patient was carefully and objectively examined prior to enrollment in the study to ensure that the patients could coordinate ABC. All of the enrolled patients had good cardiopulmonary function with Karnofsky performance scores (KPS) ranging from 80–90. None of the patients had communication barriers with the administrators of this study, and the breath holding time in mDIBH with ABC for all patients reached 30 s. This study was approved by the Research Ethics Board of the Shandong Cancer Hospital, and all patients agreed to the conditions of this trial. Completed informed consent forms were obtained from each patient.

### Computed tomography (CT) simulation

For each patient, two serious CT scans were performed using a Philips Brilliance CT Big Bore (Phillips Medical Systems, 96 Highland Heights, OH, USA). For mDIBH, CT scans were performed using the Elekta ABC system (Synergy 102™, Elekta, Crawley, UK), with the trigger threshold set at 80% of the peak value of DIBH. In addition, the latter scans were acquired after scanning with FB. Furthermore, breath training was performed at least twice by each patient prior to scanning. Patients were also immobilized using a body vacuum pillow and their arms were extended above their head.

All CT scans were acquired in spiral model (pitch = 0.938, table speed = 30 mm/s, reconstruction slice thickness = 3.0 mm) and the region scanned extended from the cricothyroid membrane to the lower edge of the liver. All CT images were transmitted to a Varian Eclipse treatment planning system (TPS) (*version 8.6.15, Varian Medical Systems, Palo Alto, CA, USA*) to determine target volumes, organs at risk (OARs) contouring, and the design of RT plans.

### Gross tumor volume (GTV) and planning target volume (PTV) acquisition

An experienced radiation oncologist contoured the GTV delineated on each CT image, and this region included the tumor and pathologically involved lymph nodes. The clinical target volume (CTV) included the GTV as well as an additional 3–5 cm in the cranio-caudal direction along the esophagus in order to include the possibility of microscopic spread. The PTV for FB plans (PTV_-FB_) included the addition of a 1.5 cm margin (for internal motion and setup error) which was applied in all three dimensions to the CTV [21]. Furthermore, the PTV for the mDIBH plans (PTV_-DIBH_) included an additional 1 cm margin to account for setup variation and ABC reproducibility (i.e., uncertainty of organ position with ABC), in as much as the tumor was immobilized during breath-hold [21-23]. Healthy lung tissue was also included in the total lung volume, while GTV was excluded.

### Radiotherapy plans

Both IMRT and VMAT plans were designed for PTV_-FB_, while VMAT was designed only for PTV_-DIBH_. These plans were administered using a VARIAN Trilogy linear accelerator (*Varian Medical Systems, Palo Alto, CA, USA*), and were named IMRT_-FB_, VMAT_-FB_, and VMAT_-DIBH_, respectively. The details of each plan are as follows:

#### *IMRT*_*-FB *_*plan*

A step-and-shoot mode was applied for seven coplanar fields with gantry angles set at 0°, 51°, 102°, 153°, 204°, 255°, and 306°. The intensity level was 20 and the dose rate was 400 MU/min.

#### *VMAT*_*-FB *_*plan*

Dual coplanar whole arcs were employed. One arc was performed in a clockwise direction from 181° to 179° with a 45° collimator angle. Conversely, the second arc was performed in a counterclockwise direction from 179° to 181° with a 315° collimator angle. The two arcs were optimized simultaneously and were delivered in opposite rotation with a maximum dose rate of 600 MU/min.

#### *VMAT*_*-DIBH *_*plan*

Three partial 135° arcs were employed according to the breath holding time with mDIBH. The gantry angles of the three arcs were set to start counterclockwise from 179° to 44°, from 67° to 292°, and from 316° to 181°. Moreover, two arcs were allowed to overlap in the mediastinal region. The collimator angle was set to 45° for the first and third arcs, and at 315° for the second arc. The maximum dose rate was 600 MU/min.

All plans were optimized using a six megavolts (MV) X-ray, and the objective dose-volume parameters were identical at the beginning of optimization for the different plans, and the parameters were adjusted following optimization until the results approached an ideal setting.

A *VARIAN Triliogy* linear accelerator equipped with a Millenni-um-120 MLC (120 leaves with a resolution at isocenter of 5 mm for the inner 20 cm and 10 mm for the outer 2 × 10 cm) was used. The gantry speed was set at 4.8°/s. For all plans, the prescription dose for the PTV was 2 Gy/fraction × 30 fractions [24]. Dose constraints for complications included minimizing doses for health lung (named total lung), heart, and spinal cord, while maintaining optimal target coverage and dose uniformity to the target volumes. In addition, the mean PTV dose was normalized to 100% of the iso-dose line. All dose distributions were computed using an analytical anisotropic algorithm available in the Eclipse planning system, and the dose grid resolution was 2 mm.

### Plan evaluations

The irradiation doses needed to target 1% and 99% of the PTVs (D_1%_ and D_99%_, respectively) were designated as the maximum and minimum doses, respectively.

Homogeneity index (HI) values were calculated based on the radiation dose ratio of 5% and 95% of the PTVs (D_5%_ and D_95%_, respectively), e.g., HI = D_5%_/D_95%_.

Conformality index (CI) values were calculated according to the *Van’t Riet* definition: CI = (TV_RI_/TV) × (TV_RI_/V_RI_), where TV_RI_ is the target volume covered by the reference isodose, TV is the target volume, and V_RI_ is the volume of the reference isodose [25]. CI and HI values are ideal when they approach a value of 1.

Comparisons were made between the different treatment plans in regard to mean irradiation dose, the percentage of the x Gy radiation dose applied to a volume of healthy tissue (V_xGy_), including for the lung (e.g., V_5_, V_10_, V_20_, V_30_, and V_40_) and heart (e.g., V_20_, V_30_, and V_40_), the maximum radiation dose applied to the spinal cord, MUs, and treatment time.

### Statistical analysis

All results are reported as the average ± standard deviation ($\bar{X}\pm S$) and SPSS 16.0 software (IBM, Chicago, IL, USA) was used for all statistical analyses. A paired *t*-test was used to compare total lung and heart volumes, GTV, and PTV for each breath status. One-way analysis of variance (ANOVA) was used to compare indices among different plans, and the difference between two plans was compared using Bonferroni multiple comparison test. Differences were considered statistically significant when the *p*-value was less than 0.05.

## Results

### Comparison of GTV, PTV, total lung and heart volumes

As shown in Table 1, the mean GTV volume determined with FB was 11.75%, and this was greater than that determined with mDIBH. However, this difference was not statistically significant (*p* > 0.05). In contrast, mean PTV and heart volume decreased (31.61% and 19.85%, respectively), while mean total lung volume increased (52.54%), with mDIBH. Furthermore, each of those differences were statistically significant (*p* < 0.05).

**Table 1:** **Target volumes and OAR volumes for FB and DIBH CT techniques (cm**^**3**^**)**

**Volume**	**FB (cm**^**3**^**)**	**DIBH (cm**^**3**^**)**	***T***	***p***
GTV	52.58 ± 19.72	46.40 ± 20.78	0.610	0.552
PTV	396.11 ± 72.43	270.91 ± 87.02	2.259	0.040
Total lung	4039.40 ± 79.86	6161.60 ±80.85	-5.282	0.000
Heart	621.08 ± 66.84	497.80 ± 86.58	3.188	0.007

*GTV* Gross tumor volume, *PTV* Planning target volume.

### Dose distribution of PTV

The D_1%_ and D_99%_ were similar among the IMRT_-FB_, VMAT_-FB_ and VMAT_-DIBH_ plans (*p* > 0.05). The CI and HI values for VMAT_-DIBH_ were slightly worse than those for IMRT_-FB_ and VMAT_-FB_, and these differences were not statistically significant (*p* > 0.05). Meanwhile, CI and HI values were similar for the IMRT_-FB_ and VMAT_-FB_ plans (*p* > 0.05).

### Organs at risk (OARs)

The VMAT_-DIBH_ plan significantly reduced the mean dose and the V_5_ to V_40_ values for total lung compared with the IMRT_-FB_ and VMAT_-FB_ plans (except for V_5_, as shown in Table 2) (*p* < 0.05). The V_10_ of VMAT_-FB_ was also higher than that of the IMRT_-FB_ plan, yet the difference was not significant (*p* > 0.05). The mean dose, V_5_, V_20_, V_30_, and V_40_ were also similar for the IMRT_-FB_ and VMAT_-FB_ plans (*p* > 0.05).

**Table 2:** Dose-volume indices of the PTV and OARs

**Variable**	**IMRT**_**-FB**_	**VMAT**_**-FB**_	**VMAT**_**-DIBH**_	***F***	***p***
PTV					
D_1%_ (Gy)	65.67 ± 0.41	65.74 ± 0.35	66.10 ± 0.44	2.599	0.098
D_99%_ (Gy)	58.82 ± 0.52	58.76 ± 0.51	58.35 ± 0.41	2.156	0.141
CI	0.90 ± 0.03	0.90 ± 0.03	0.86 ± 0.04	2.864	0.079
HI	1.05 ± 0.02	1.06 ± 0.02	1.07 ± 0.03	2.485	0.107
Total lung					
V_5_ (%)	83 ± 9	83 ± 9	74 ± 10	2.274	0.128
V_10_ (%)	64 ± 9	70 ± 10	56 ± 10	4.808	0.019
V_20_(%)	26. ± 5	24 ± 6	18 ± 4	5.518	0.012
V_30_ (%)	8 ± 4	8 ± 3	4 ± 2	4.062	0.032
V_40_ (%)	4 ± 2	4 ± 1	2 ± 0	3.790	0.039
D_mean _(Gy)	15.45 ± 2.24	15.30 ± 2.19	12.57 ± 1.78	4.884	0.018
Heart					
V_20 _(%)	71 ± 2.46	61 ± 2.77	61 ± 2.73	0.336	0.719
V_30 _(%)	38 ± 2.37	36 ± 2.35	35 ± 2.20	0.032	0.968
V_40_ (%)	22 ± 1.42	20 ± 1.40	19 ± 1.25	0.097	0.908
D_mean_	28.83 ± 8.60	28.82 ± 8.71	27.32 ± 8.53	0.080	0.923
Spinal cord					
D_max_(Gy)	43.54 ± 2.62	44.83 ± 2.19	41.25 ± 4.40	2.542	0.103
Monitor units	798.38 ± 11.20	508.25 ± 6.49	506.88 ± 7.88	29.156	0.000
Treatment time (s)	367.64 ± 3.39	170 ± 0	124 ± 0	349.997	0.000

*CI* Conformality index, *HI* Homogeneity index.

For the heart, the V_20_, V_30_, and V_40_ values were similar between the VMAT_-DIBH_ and VMAT_-FB_ plans (*p* > 0.05), and these values were lower than those of the IMRT_-FB_ plan. The mean irradiation dose for the heart was also similar between the IMRT_-FB_ and VMAT_-FB_ plans, and these doses were higher than that for the VMAT_-DIBH_ plan.

There were no significant differences in the D_max_ values for the spinal cord among the plans (*p* > 0.05).

### MUs and delivery time

MUs for the IMRT_-FB_ plan (798.38 ± 112.99) were significantly greater than those for the VMAT_-FB_ (508.25 ± 64.95) and VMAT_-DIBH_ (506.88 ± 78.87) plans (*p* < 0.05). The estimated treatment time (from first filed beam-on to the last filed beam-off) for VMAT_-DIBH_ (124 ± 0 s) was also shorter than that for VMAT_-FB_ (170 ± 0 s), and was also significantly shorter than the measured treatment time for IMRT_-FB_ (367.64 ± 33.90 s) (*p* < 0.05).

## Discussion

Previously, VMAT has been shown to achieve better dose delivery with a shorter treatment time and fewer MUs [10-20]. However, VMAT has also been associated with a larger low dose region that affects OARs in some cases [10-17]. Correspondingly, differences in lung V_10_ between the VMAT_-FB_ and IMRT_-FB_ plans in the present study were observed.

Treatment-related pneumonitis represents critical radiation toxicity during RT for thoracic EC, and dose-volume indices are commonly used to predict this side effect [26-28]. While V_20_, V_30_, and the mean lung dose have been considered classic predictors of treatment-related pneumonitis, the predictive value of V_5_, V_10_, and V_13_ have also been considered [29-31]. Therefore, the approach which reduces lung dose for all dose-volume indices, provides sufficient PTV coverage, and achieves perfect dose distribution, is preferred [32]. Accordingly, VMAT represents a preferred method based on its shorter treatment time, fewer MUs, and better dose delivery. Furthermore, in association with ABC, VMAT may potentially spare a greater region of normal tissue from damage [33].

The displacement and deformation of thoracic EC caused by breath motion can significantly affect the precision of RT. In addition, the randomness of breath phase at the scanning moment with FB can potentially introduce more uncertainty. This is usually compensated for with larger margins. However, if the margins are extended too far, this can increase the probability that radiation injury will occur. Accordingly, the ability to reduce breath motion is important, especially for patients with distal EC. The application of ABC with mDIBH was found to further reduce lung radiation dose by increasing the absolute lung volume.

At the beginning of this study, IMRT and ABC were combined for RT of EC. However, the feasibility of this approach was limited by the longer time needed for each field and fraction of IMRT. Each IMRT plan has seven fields, and sometimes one field may be divided into more than one subfield, potentially leading to 14 or more subfields. In addition, patients need to rest for at least six intervals, and this may compromise the reproducibility of the breath hold motion due to muscle fatigue. Second, if one field or subfield is not completed within the breath-holding time of the patient, it is difficult to ensure that the relative position of the MLC and PTV will be identical to the moment of beam off. Regarding the VMAT plans, completing a whole arc using segments was not feasible, and this approach would waste time and increase uncertainty [33]. Therefore, duration of treatment for each field and fraction, as well as the risk of secondary tumors for patients who survive for an extended period of time, are important factors to consider. In addition, for the whole arc(s) VMAT plans, a larger low dose region affecting OARs was observed, although good dose delivery was achieved in a shorter period of time and with fewer MUs using these plans.

It was observed that the VMAT_-DIBH_ plans with three 135° arcs were associated with lower CI values than the IMRT_-FB_ and VMAT_-FB_ plans, although the differences were not significant. A possible reason for this phenomenon is that VMAT_-DIBH_ involves fewer control points than the other plans. Thus, another arc < 135° may be needed to raise CI values [34]. For the VMAT_-FB_ plan, it contained 3–4 arcs (including 2–3 rest times for the patient), which is reasonable to ensure the reproducibility and stability of the breath hold motion based on our experience with RT and ABC applied to breast cancer, lung cancer, and hepatocellular carcinoma.

ABC has been shown to be an effective approach for improving the precise application of RT for thoracic and abdominal cancer. Specifically:

(1) ABC facilitates the positioning of the target volume of a tumor by limiting the motion of the target volume induced by respiration. Consequently, margins between the CTV and the PTV were able to be reduced by effectively restricting respiratory motion. Moreover, OARs were able to be spared by ensuring accurate positioning of the tumor [33].

(2) DIBH can increase lung volume, thereby sparing normal lung tissue during treatment. Moreover, the use of ABC to improve DIBH has previously been applied to RT for breast cancers, lung cancers, and lymphomas, with positive results reported [35].

(3) The use of ABC to achieve varying degrees of breath status, including DIBH, deep expiratory hold (DEBH), or mDIBH, potentially changes the spatial relationship between tumor and OARs, and this may subsequently affect the dose-volume of OARs.

In this study, the lower radiation dose for lung tissue can be attributed to the reduction in PTV margins and the increase in lung volume with DIBH. Total lung volume and dose-volume parameters for the lung are two important factors [36]. Displacement of the ECs in different directions was not able to be clearly determined in the present study, although it is possible to measure these parameters using 4D-CT. In simulations, respiratory phase randomness was associated with too much uncertainty regarding GTV_FB_, and this uncertainty was effectively reduced with the use of DIBH. Furthermore, the 5 mm reduction in tumor margins was found to be moderate and reasonable in the present study [20,21], and dosimetric benefit was maintained in relation to lung volume if the same margins were applied for PTV_-FB_ and PTV_-DIBH_. Heart volume with mDIBH was also significantly smaller than with FB, while the dose-volume indices for VMAT_-DIBH_ were lower than those of IMRT-_FB_ and VMAT_-FB_. These results were very promising, although the dose-volume indices did not differ significantly. This may be due to the spatial limitation of heart beats during DIBH, reductions in tumor margins, and the increased distance for PTV.

Overall, VMAT combined with ABC in the present study achieved good, accurate dose distribution, involved a shorter treatment time, and reduced low dose exposure of OARs. The total treatment time was approximately 115 s, which includes the rest time for the patient and the gantry rotation time for beam preparation. Moreover, this period of time is shorter than that needed to complete double whole arc VMAT. CI values can also increase with low speed gantry rotation, and this speed should be as fast as possible, as soon as possible, in order to reduce the uncertainty of RT, especially when applying ABC [37].

The prescription dose used in this study was 60 Gy/30 F, and was selected based on survival rates versus escalating dose. In addition, another radiation therapy oncology group (RTOG) had previously demonstrated that a dose of 50.4 Gy was associated with an acceptable clinical outcome [38,39]. In particular, lung irradiation dose was found to be the major limiting factor for increasing the PTV dose. However, using VMAT with ABC for mDIBH, the prescription dose could be higher. Although the dosimetric benefits were evaluated by comparing treatment strategies, the reproducibility of these results need to be confirmed, particularly regarding the clinical benefit of the breath hold motion. However, clinical outcome may not be obtained in a short period of time. Therefore, incorporation of image-guided RT could further improve the precision of the currently proposed method without significantly increasing treatment time. In addition, patients treated in the present study will continue to be monitored in order to verify the current results and to improve the design of future studies.

## Conclusions

VMAT combined with ABC for mDIBH achieved a similar dose distribution for tumor targets and spared OARs to a greater extent than IMRT and double whole arc VMAT with FB. Therefore, VMAT combined with ABC for mDIBH is recommended for thoracic EC patients with good cardiopulmonary function.

## Competing interests

The authors declare that they have no competing interests.

## Authors’ contributions

All authors read and approved the final manuscript. Guanzhong Gong, Ruozheng Wang, Yujie Guo, Deyin Zhai, Tonghai Liu, Jie Lu and Jinhu Chen carried out the design and optimization of IMRT and VMAT treatment plans, and Chengxin Liu carried out the statistics of this study, and Yong Yin administrated the study. All authors participated in the sequence alignment and drafted the manuscript.
